# The effects of exercise interventions on bone mineral density in middle-aged and older men: a systematic review and meta-analysis

**DOI:** 10.3389/fphys.2026.1825374

**Published:** 2026-05-29

**Authors:** Chengqian Feng, Jie Shu, Yang Li, Zhicheng Lin

**Affiliations:** 1 Department of Physical Education, Xiamen University, Xiamen, China; 2 Department of Physical Education, Peking University, Beijing, China; 3 Zhongshan College, Dalian Medical University, Dalian, China

**Keywords:** bone mineral density, exercise, men, meta-analysis, osteoporosis, training

## Abstract

**Introduction:**

Exercise interventions are increasingly used as a non-pharmacological strategy to promote skeletal health. Nevertheless, their site-specific effects on bone mineral density (BMD) in middle-aged and older men remain unclear and inconsistent. This study aimed to systematically assess the impacts of exercise interventions on BMD across multiple skeletal sites in this population.

**Methods:**

We performed a comprehensive systematic search in major electronic databases (PubMed, Web of Science, Embase) to identify randomized controlled trials investigating exercise and BMD in men. Eligible studies were selected according to prespecified inclusion criteria, and a random-effects model was applied in the meta-analysis to compute pooled effect sizes.

**Results:**

Pooled analyses revealed that exercise interventions significantly increased BMD at the lumbar spine and femoral neck in middle-aged and older men. However, no significant beneficial effects were detected at the total hip or other cortical-dominant skeletal regions. Subgroup analyses further confirmed that a training frequency of ≥f sessions per week, an intervention duration of >6 months, and multicomponent exercise regimens maximized skeletal benefits.

**Discussion:**

Exercise represents an effective approach to improve site-specific BMD in aging men, especially at high-fracture-risk sites including the lumbar spine and femoral neck. These results offer evidence-based implications for designing sex-specific exercise prescriptions, and underscore the key roles of training frequency and exercise modality in eliciting optimal skeletal adaptive responses.

**Systematic Review Registration:**

https://www.crd.york.ac.uk/prospero/, identifier CRD420251126500.

## 1 Introduction

Osteoporosis is a systemic skeletal disorder, defined by the World Health Organization (WHO) as a bone mineral density value 2.5 or more standard deviations below the mean of healthy young adults (NIH Consensus Development Panel on Osteoporosis Prevention, 2001; Organization, 2003). With the aging of the global population, the socioeconomic burden of osteoporosis is projected to increase further (Rachner et al., 2011). In recent years, male osteoporosis has gained increasing attention from researchers. Epidemiological surveys indicate a high prevalence among men, averaging approximately 12% globally and exceeding 20% in some regions (Chen et al., 2016; Salari et al., 2021; Zamani et al., 2018; Fuggle et al., 2024), and once fractures occur, disability and mortality rates may even surpass those observed in women (Bliuc et al., 2009). Nevertheless, most osteoporosis research has focused on women, leading to underestimation, underdiagnosis, and undertreatment in men (Rinonapoli et al., 2021; Qaseem et al., 2017). Current clinical guidelines for managing male osteoporosis are largely extrapolated from studies conducted in female cohorts, which overlooks the specific mechanosensitivity of the male skeleton (Rinonapoli et al., 2021). Unlike the rapid bone loss in postmenopausal women characterized by trabecular disconnection, age-related bone loss in men is primarily driven by trabecular thinning and compensatory periosteal expansion (Emmert et al., 2024).

In clinical practice, pharmacologic therapies, such as calcium, vitamin D, hormone replacement therapy, and bisphosphonates, are commonly used to treat osteoporosis. However, these regimens often entail prolonged treatment courses, potential adverse effects, high costs, and suboptimal adherence (Fuggle et al., 2024). As a non-pharmacologic approach, exercise interventions have shown beneficial effects in maintaining and improving BMD (Fuggle et al., 2024; Zhang et al., 2022).

However, current studies investigating whether exercise improves BMD in men are limited in number and inconsistent in their findings, with some studies reporting significant benefits while others show no significant effects. These conflicting results necessitate a more rigorous meta-analysis to clarify the actual efficacy of exercise interventions in this population (Hamilton et al., 2022; Lu et al., 2023). To address this knowledge gap, this study systematically synthesizes available evidence through a meta-analysis to quantitatively assess the impact of exercise on BMD in this specific population. Our findings aim to provide a robust evidence base for the development of sex-specific exercise prescriptions and to optimize osteoporosis prevention strategies for aging men.

## 2 Methods

This systematic review and meta-analysis was prospectively registered with the International Prospective Register of Systematic Reviews (PROSPERO) under registration number CRD420251126500. The review was conducted in accordance with the registered protocol, with no major deviations or amendments made during the study period.

### 2.1 Study selection

**Figure 1:**
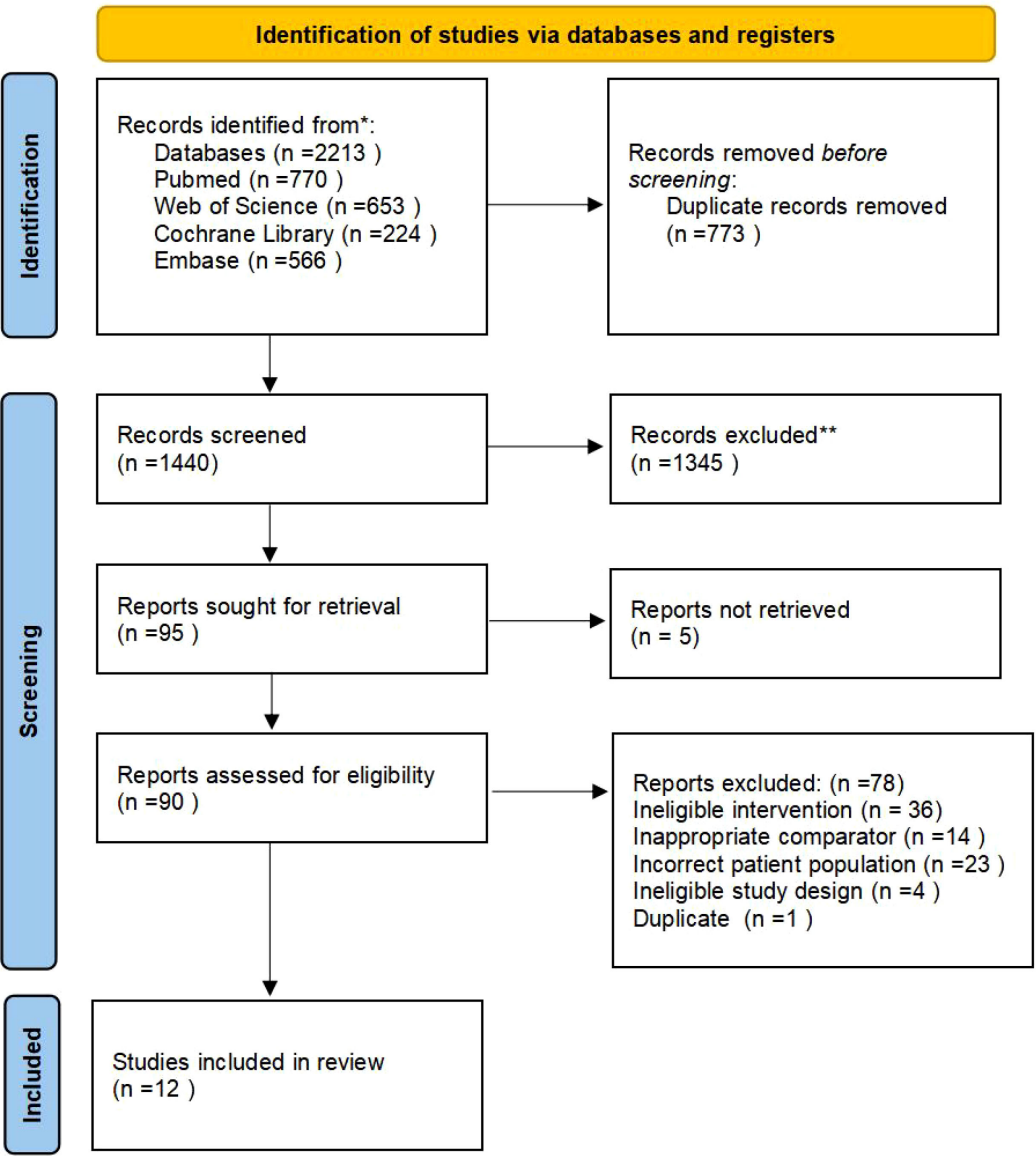
PRISMA flow diagram.

The initial database search yielded 2,213 records. After removing 773 duplicates, 1,440 titles and abstracts were screened, and 90 full-text reports were formally assessed for eligibility. Ultimately, 12 studies met the inclusion criteria and were included in the systematic review (**Figure 1**). The primary reasons for exclusion during the full-text assessment included ineligible interventions (n=36), incorrect patient populations (n=23), and inappropriate comparators (n=14).

### 2.2 Search strategy

Two investigators searched electronic databases and screened the reference lists of related studies. First, MEDLINE (PubMed), Cochrane CENTRAL, Embase, Web of Science, Scopus, and CNKI, Wanfang, VIP, SinoMed were searched from inception to December 20, 2025. Search strings combined controlled vocabulary and free-text terms for population, intervention, and outcome, for example: (“male” OR “men”) AND (“exercise” OR “training” OR “resistance” OR “weight-bearing” OR “impact”) AND (“osteoporosis” OR “bone mineral density” OR “BMD”). Database-specific adaptations were also applied. Second, the reference lists of eligible studies and prior reviews were hand-searched to identify additional articles. Searches were limited to human, peer-reviewed, randomized, or semi-randomized trials in English or Chinese; gray literature (e.g., dissertations, proceedings, reports) was excluded. The complete search strategy is presented in **Table 1**.

**Table 1:** Search strategy.

#1	((Exercise[MeSH Terms] OR “Exercise”[Title/Abstract] OR “Physical Activity”[Title/Abstract] OR “Resistance Training”[Title/Abstract] OR “Strength Training”[Title/Abstract] OR “Weight-bearing”[Title/Abstract] OR “Training”[Title/Abstract] OR “Exercise Therapy”[MeSH Terms] OR “Sport”[Title/Abstract] OR “Aerobic”[Title/Abstract])) AND ((“Bone Density”[MeSH Terms] OR “Bone Density”[Title/Abstract] OR “Bone Mineral Density”[Title/Abstract] OR “BMD”[Title/Abstract] OR “Bone Mineral Content”[Title/Abstract] OR “BMC”[Title/Abstract] OR “Osteoporosis”[Title/Abstract] OR “Osteopenia”[Title/Abstract] OR “Bone Mass”[Title/Abstract])) AND ((“Men”[MeSH Terms] OR “Male”[MeSH Terms] OR “Men”[Title/Abstract] OR “Male”[Title/Abstract] OR “Older”[Title/Abstract] OR “Elderly”[Title/Abstract] OR “top-aged”[Title/Abstract] OR “Aging”[Title/Abstract] OR “Senior”[Title/Abstract]))
#2	(“Bone Density”[MeSH Terms] OR “Bone Density”[Title/Abstract] OR “Bone Mineral Density”[Title/Abstract] OR “BMD”[Title/Abstract] OR “Bone Mineral Content”[Title/Abstract] OR “BMC”[Title/Abstract] OR “Osteoporosis”[Title/Abstract] OR “Osteopenia”[Title/Abstract] OR “Bone Mass”[Title/Abstract])
#3	(“Men”[MeSH Terms] OR “Male”[MeSH Terms] OR “Men”[Title/Abstract] OR “Male”[Title/Abstract] OR “Older”[Title/Abstract] OR “Elderly”[Title/Abstract] OR “top-aged”[Title/Abstract] OR “Aging”[Title/Abstract] OR “Senior”[Title/Abstract])
#4	(randomized controlled trial[pt] OR controlled clinical trial[pt] OR randomized[tiab] OR placebo[tiab] OR “clinical trials as topic”[mesh:noexp] OR randomly[tiab] OR trial[tiab])
#5	(animals[mesh])
#6	#1 AND #2 AND #3 AND #4 AND
#7	#6 NOT #5

### 2.3 Study selection

Inclusion criteria were: (a) middle-aged and older men (≥45 years); trials enrolling both sexes were eligible when male-specific data were extractable; (b) intervention groups receiving structured exercise; (c) comparator groups receiving usual care or placebo activity (e.g., low-intensity stretching); (d) reporting BMD at the lumbar spine, total hip, or femoral neck by DXA or QCT; and (e) parallel-group randomized or semi-randomized controlled trials with outcome data.

Exclusion criteria were: (a) animal studies; (b) Pharmacological interventions that directly confound BMD; (c) weight-loss trials not involving exercise as the primary intervention; (d) studies reporting only biomarkers without BMD; (e) non-randomized designs (case reports, single-arm studies, cohort without control).Two reviewers independently screened abstracts and full texts against the criteria; disagreements were resolved by discussion with a third reviewer.

### 2.4 Data extraction

Two independent researchers extracted data in a standardized form and cross-checked the results. The extracted items included: (a) bibliographic data (first author, year, country); (b) participant characteristics (sample size, mean age, BMI where available); (c) intervention details (modality, frequency, session duration, total duration) and comparator; and (d) outcomes (BMD at lumbar spine, total hip, femoral neck; measurement method; follow-up time points). When trials had multiple arms, data were recorded for the exercise versus appropriate control comparison specified by the review question. When necessary, numeric values were estimated from figures using standard methods or requested from the authors.

### 2.5 Assessment of risk of bias

Methodological quality was assessed using the Cochrane Risk of Bias (RoB) 2.0 tool by two independent reviewers across the following domains: randomization process, allocation concealment, deviations from intended interventions, missing outcome data, outcome measurement, and selective reporting. Each domain was judged as low risk, some concerns, or high risk following the tool’s signaling questions; an overall judgment was derived accordingly. Disagreements were resolved through discussion.

### 2.6 Data synthesis and statistical analysis

Effect sizes for continuous outcomes were calculated as mean differences (MD) with 95% confidence intervals (CI), since all included studies reported BMD using consistent units. When multiple valid measures for the same outcome were available at the same skeletal site, post-intervention values were preferentially used for analysis; change scores were adopted only when exclusively reported. For trials with multiple comparable exercise intervention arms, groups were combined into a single pairwise comparison to avoid unit-of-analysis errors. Studies without sufficient quantitative data, even after attempting to contact the original authors, were excluded from the meta-analysis (Higgins et al., 2003). Statistical heterogeneity was assessed using the chi-squared test and the I^2^ statistic, with substantial heterogeneity defined as p<0.05 or I^2^>50% (Borenstein et al., 2017). Before exploring potential sources of heterogeneity, sensitivity analyses were performed using leave-one-out methods and model diagnostics to verify the robustness of pooled estimates and identify any individual study with an excessive impact on the overall results.

Subgroup analyses were then conducted based on three prespecified factors: intervention duration (ur vs. >6 months), weekly training frequency (<3 vs. ≥s sessions/week), and exercise modality (single vs. multiple), age (<65 vs. ≥s. years). Between-subgroup differences were formally examined by comparing effect sizes and changes in heterogeneity. The overall treatment effect was consistent across all subgroups, with no significant between-group differences observed (P>0.05).

Funnel plots and Egger’s test were employed to evaluate small-study effects bias, with statistical significance set at p < 0.05 to indicate potential asymmetry. All primary analyses were performed using Stata version 18.0. The GRADE approach was planned to assess the certainty of evidence for the main comparisons, considering the risk of bias, inconsistency, indirectness, imprecision, and publication bias, and rating the overall certainty as high, moderate, low, or very low.

## 3 Results

### 3.1 Study selection

The initial database search yielded a total of 2,213 records. After removing 773 duplicates, 1,440 titles and abstracts were screened, leading to the exclusion of 1,345 records that did not meet the inclusion criteria. Ultimately, 12 original studies were included in this systematic review and meta-analysis (**Figure 1**).

### 3.2 Study characteristics

Twelve trials were included (Yanmei et al., 2017; Harding et al., 2020; Woo et al., 2007; Stewart et al., 2005; Bjerre et al., 2019; Kukuljan et al., 2011; Mohamed et al., 2018; Naderi et al., 2021; Kim et al., 2018; Bolam et al., 2015; Whiteford et al., 2010; Helge et al., 2014), enrolling 1061 participants (540 in the exercise arms and 521 in the control arms). Five trials used a two-arm parallel design (Harding et al., 2020; Woo et al., 2007; Kukuljan et al., 2011; Bolam et al., 2015; Helge et al., 2014); the remainder followed comparable single-comparison designs, as specified by each study. The publication years ranged from 2007 to 2021. The mean participant age ranged from 50.9 to 77.8 years, and most trials compared an exercise intervention with usual care/no intervention. Two trials combined exercise with nutritional co-interventions (Kukuljan et al., 2011). Seven trials implemented multicomponent interventions. Resistance training was the most common intervention (Harding et al., 2020; Woo et al., 2007; Stewart et al., 2005; Bjerre et al., 2019; Kukuljan et al., 2011; Mohamed et al., 2018; Kim et al., 2018; Bolam et al., 2015; Whiteford et al., 2010). Impact/weight-bearing exercise appeared in seven trials (Harding et al., 2020; Woo et al., 2007; Bjerre et al., 2019; Kukuljan et al., 2011; Naderi et al., 2021; Kim et al., 2018; Bolam et al., 2015; Helge et al., 2014) and aerobic training in two (Stewart et al., 2005; Mohamed et al., 2018). Sport-based programs included football (Bjerre et al., 2019; Helge et al., 2014) and table tennis (Naderi et al., 2021). Other modalities included whole-body vibration (Yanmei et al., 2017) and Tai Chi (Woo et al., 2007). The intervention duration ranged from 6 to 18 months. Most trials prescribed 2–5 sessions/week, 20–60 min/session. The baseline and design characteristics are summarized in **Tables 2** and **3**, respectively.

**Table 2:** Basic characteristics.

Study	Study design	Area	n(EG/CG)	Age(EG/CG)	BMI(EG/CG)	Outcomes	Device
Mohamed et al. (2018)	RCT	Egypt	25/25	50.9 ± 5.0	26.34 ± 2.39	①②	OsteoSys PRIMUS
Bjerre et al. (2019)	RCT	Denmark	109/105	68.4 ± 6.2	NR	①②③④	NR
Bolam et al. (2015)	RCT	Australia	13/14	62.1 ± 6.9/58.6 ± 7.4	26.6 ± 3.6/26.6 ± 3.4	①②③④⑤	Hologic Discovery W
			15/14	59.3 ± 5.7/58.6 ± 7.4	25.8 ± 2.8/26.6 ± 3.4		Hologic Discovery W
Harding et al. (2020)	semi-RCT	Australia	34/26	67.1 ± 7.5	26.7 ± 3.5	①②③④	Medix DR
			33/26		26.7 ± 3.5		
Helge et al. (2014)	RCT	Denmark	9/8	68.2 ± 3.2	26.1 ± 3.9/27.9 ± 4.6	③⑤⑥	iDXA (Lunar Corporation, Madison, WI, USA)
			9/8		27.4 ± 2.8/27.9 ± 4.6	①②	
Kemmler et al. (2020)	RCT	German	21/22	77.8 ± 3.6/79.2 ± 4.7	25.0 ± 3.0/24.5 ± 1.9		Somatom Force CT,Hologic QDR 4500
Kim et al. (2018)	RCT	Korea	23/18	70.8	NR	①②③	QDR-4500A (Lunar Prodigy Advance GE Lunar
Kukuljan et al. (2011)	RCT	Australia	46/44	61.7 ± 7.6/61.7 ± 7.7	27.4 ± 3.7/27.7 ± 3.3	①②③	Prodigy DXA, GE Lunar Corporate (Madison, WI, USA)
			45/45	60.7 ± 7.1/59.9 ± 7.4	28.1 ± 3.3/26.7 ± 2.9		
Naderi et al. (2021)	RCT	Iron	16/20	66.3 ± 3.6/67.0 ± 3.9	27.1 ± 1.5/26.9 ± 1.8	①③④	Prodigy DXA, GE Lunar Corporate (Madison, WI, USA)
Yanmei et al. (2017)	RCT	China	18/18	63.67 ± 1.63/64.33 ± 2.16	NR	①③	NORLAND XR-80 (USA)
Stewart et al. (2005)	RCT	USA	25/26	61.7 ± 4.5/63.59	29.7 ± 3.0/29.7 ± 3.8	①②③④⑥	GE Lunar Prodigy
Whiteford et al. (2010)	RCT	Australia	61/66	64 ± 6	26.4 ± 3.1/26.3 ± 3.0	①②③④	Lunar Prodigy DPX-L (Lunar Corporation, Madison, WI, USA)
Woo et al. (2007)	RCT	China	30/29	68.2 ± 2.4/68.07 ± 3.0	23.56 ± 3.4/23.89 ± 3.1	②	Hologic QDR 4500
			29/29	68.67 ± 3.0/68.07 ± 3.0	24.10 ± 3.4/23.89 ± 3.1		

EG, exercise group; CG, control group; ①, lumbar spine BMD; ②, total hip BMD; ③, femoral neck BMD; ④, greater trochanter BMD;⑤whole body BMD; ⑥, femoral shaft BMD.

**Table 3:** Intervention characteristics.

Study	Interventions		Cycle	Frequency/wk	Time	Supervised	Dropout	ITT	Advertise event
	EG	CG							
Mohamed et al. (2018)	AT+RT+BT+PL	PL	1year	3	1h	yes	12%(3)/20%(5)	yes	NR
Bjerre(2019)	F	UC	6 months	2	1h	yes	4.76%(5)/4.59%(5)	yes	Achilles tendon tear、fracture
Bolam et al. (2015)	HI	UC	9months	4	1h -	yes	23.08%(3)/7.14%(1)	yes	NR
	MOD	UC		4			13.33%(2)/7.14%(1)	yes	
Harding(2020)	HiRIT	UC	8months	2	30min	yes	11.8%(4)/19.2%(5)	yes	Fall
	IAC	UC		2			9.1%(3)/19.2%(5)	yes	Fall
Helge(2014)	F	UC	1year	2~3	1h	yes	11.1%/0	no	Achilles tendon tear, fracture
	RT	UC					0	no	NR
Kim(2018)	IT+RT+BT/CRE	PL	6months	3~5	1.5h	yes	11.5%(3)/28.0%(7)	no	
Kukuljan(2011)	IT+RT+milk	milk	18months	3	70min	yes	4.4%(2)/4.4%(2)	yes	NR
	IT+RT	UC	18months			yes	4.4%(2)/4.4%(2)	yes	
Naderi(2021)	TT	UC	6months	3~5	1.5h	yes	10%(4)/0	NR	Ankle sprain、thigh strain
Yanmei et al. (2017)	WVT	UC	6months	3	10min		NR	NR	NR
Stewart(2005)	RT+AT	UC	6months	3	1.5h	yes	10.53%(6)/8.62%(5)	no	NR
Whiteford(2010)	RT	PL	1year	3	1h	yes	21.9%(16)/7.1%(5)	yes	Hip injury
Woo(2007)	TC	UC	1year	3	1h	NR	0/3.33%(1)	no	NR
	RT	UC		3	45min		3.33%(1)/3.33%(1)	no	

EG, exercise group; CG, control group; PL, Placebo; UC, Usual care; AT, aerobic training; RT, resistance training; BT, balance training; IT, impact training; F, football; HI, High-dose impact-loading; MOD, Moderate-dose impact-loading; HiRIT, High-Intensity Resistance and Impact Training; IAC, Isometric Axial Compression Training; NS, nutrition supplement; TT, table tennis; WVT, Whole Body Vibration Training; TC, Taichi; NR, Not report.

### 3.3 Risk of bias

**Figure 2:**
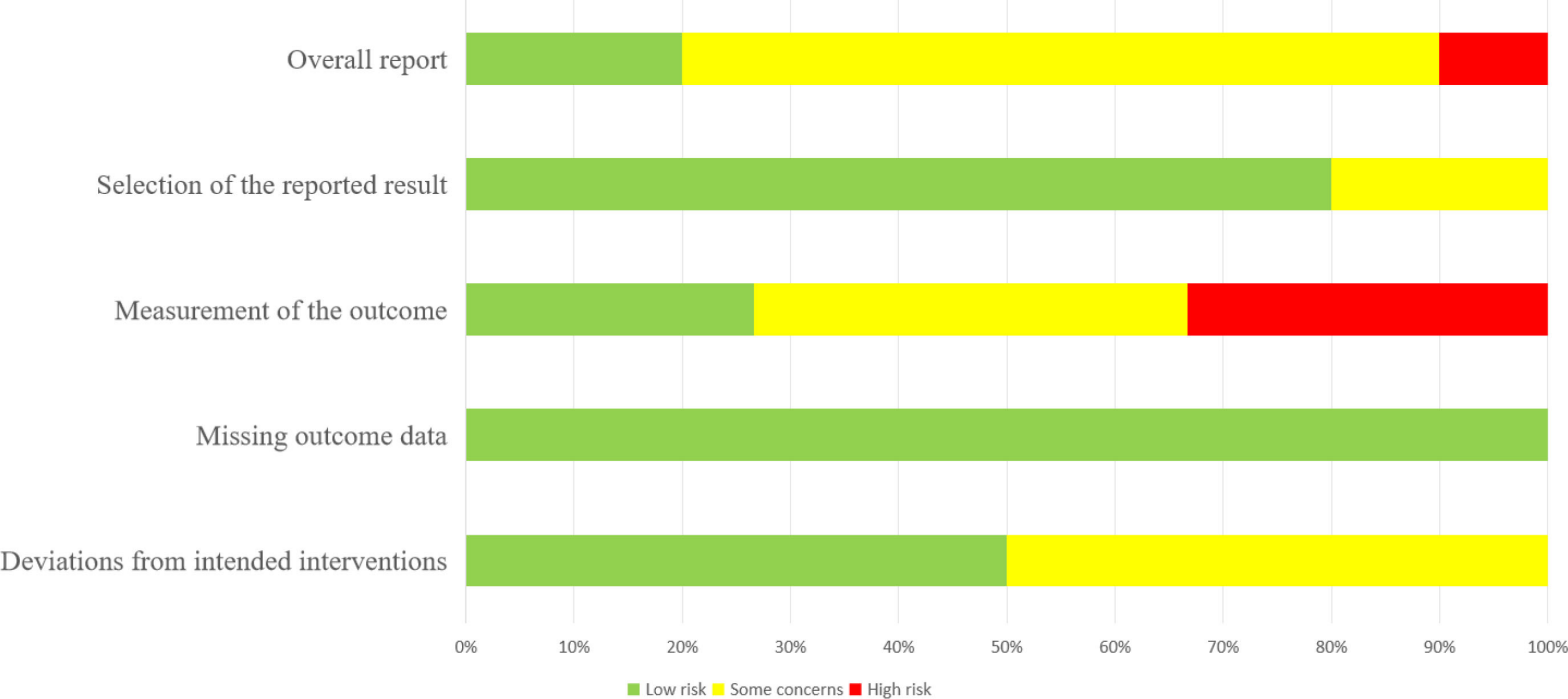
Risk of bias of included studies.

The quality assessment of the included studies revealed a generally acceptable methodological framework as shown in **Figure 2**. Notably, the majority of the studies demonstrated a high level of rigor in measurement of the outcome and missing outcome data, with 67% and 50% of trials rated as low risk of bias, respectively. While the overall bias was predominantly characterized by some concerns, it is encouraging to note that the occurrence of high risk was strictly limited across most domains. This suggests that the foundational evidence, despite some methodological nuances, remains predominantly reliable for the current synthesis.

### 3.4 Meta-analysis results

#### 3.4.1 Lumbar spine BMD

**Figure 3:**
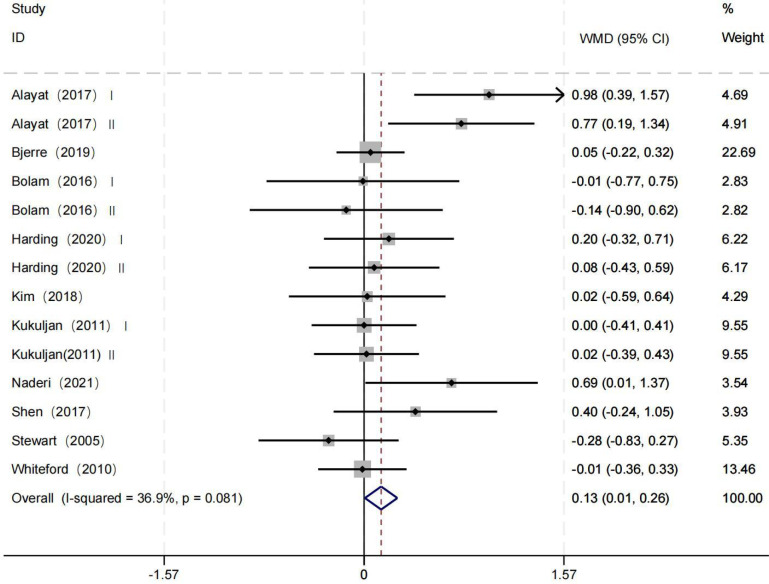
Meta-analysis results of the effect of exercise on lumbar spine BMD.

Eleven studies contributed data. Exercise was associated with a small but statistically significant improvement in lumbar spine BMD (MD = 0.13, 95% CI 0.01 to 0.26). Between-trial heterogeneity was substantial (I² = 36.9%, p = 0.081). (**Figure 3**).

#### 3.4.2 Total hip BMD

**Figure 4:**
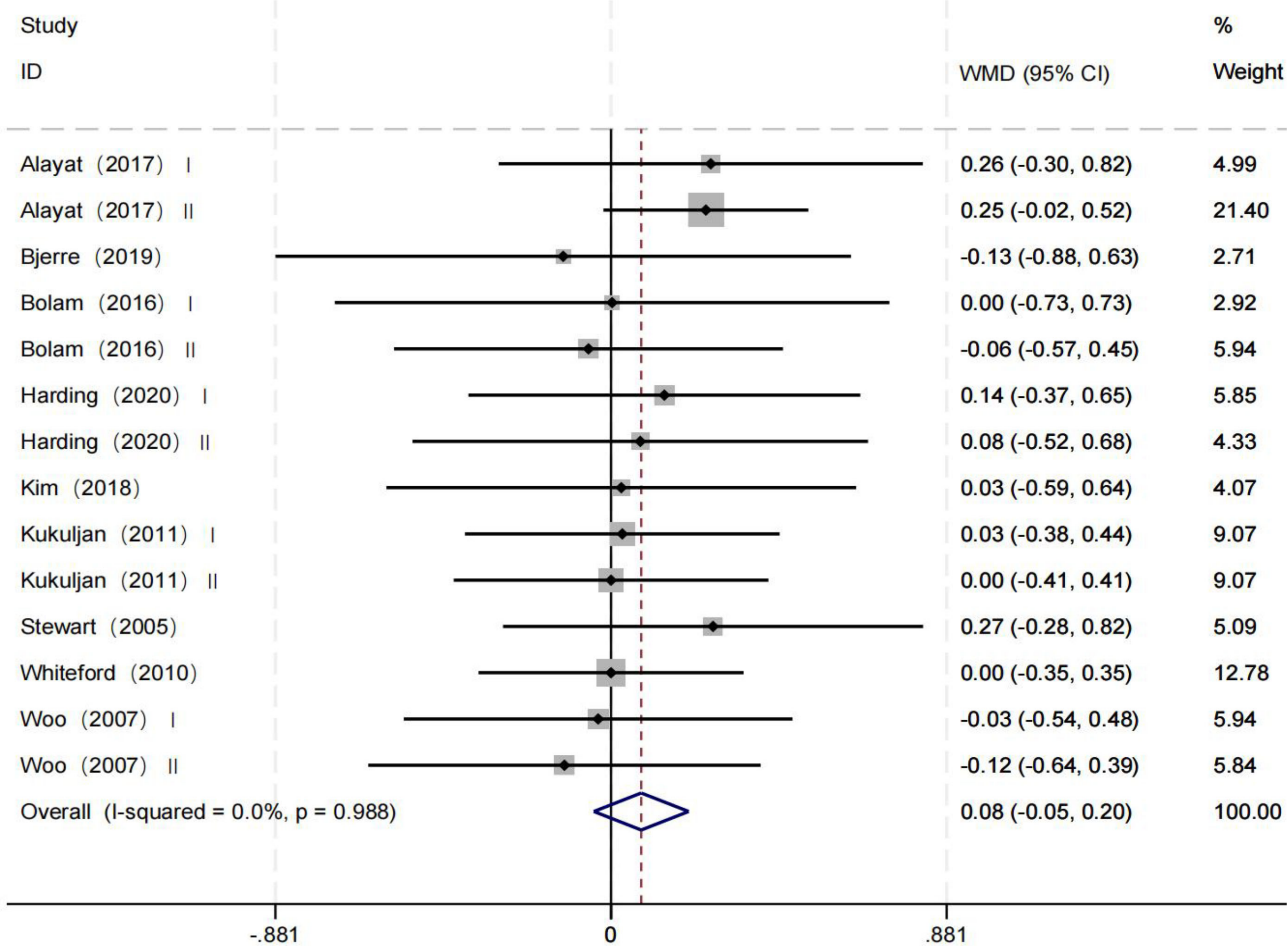
Meta-analysis results of the effect of exercise on total hip BMD.

Nine studies were included. The pooled random-effects estimate for the total hip was not statistically significant (MD = 0.08, 95% CI −0.05 to 0.20), with zero heterogeneity (I² = 0%, p = 0.988). (**Figure 4**).

#### 3.4.3 Femoral neck BMD

**Figure 5:**
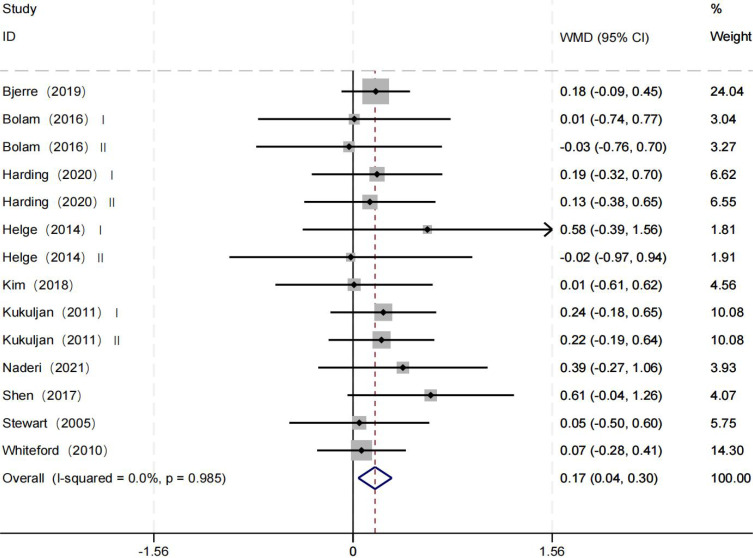
Meta-analysis results of the effect of exercise on femoral neck BMD.

Ten studies were included. Exercise produced a small but statistically significant benefit at the femoral neck (MD = 0.17, 95% CI 0.04 to 0.30), with zero heterogeneity (I² = 0%, p = 0.985). (**Figure 5**).

#### 3.4.4 Greater trochanter BMD

**Figure 6:**
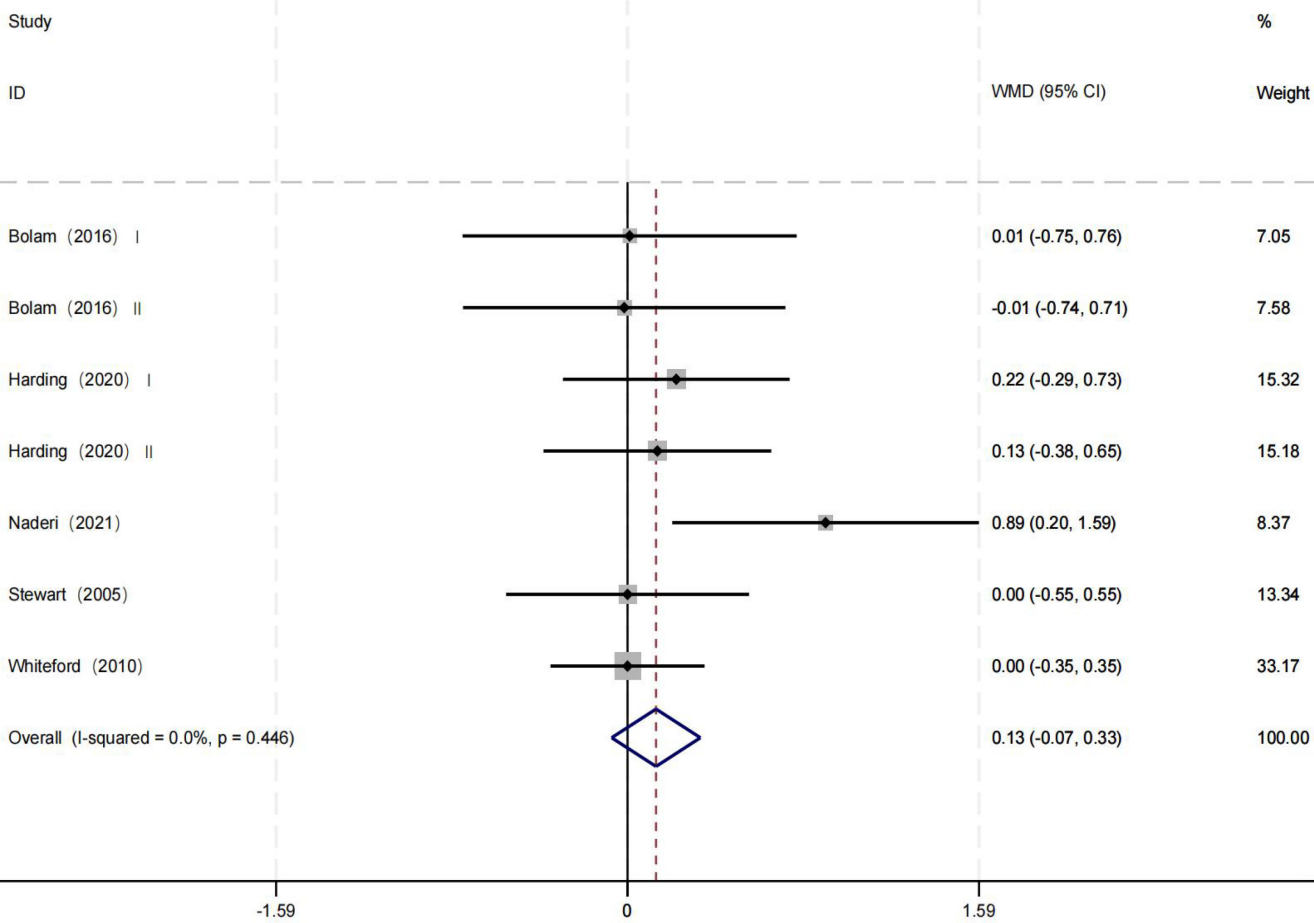
Meta-analysis results of the effect of exercise on greater trochanter BMD.

Seven studies were included in this review. Exercise showed a mildly improvement but no significant effect on greater trochanter BMD (MD = 0.13, 95% CI −0.07 to 0.33), with zero heterogeneity (I² = 0%, p = 0.446). (**Figure 6**).

#### 3.4.5 Whole body BMD

**Figure 7:**
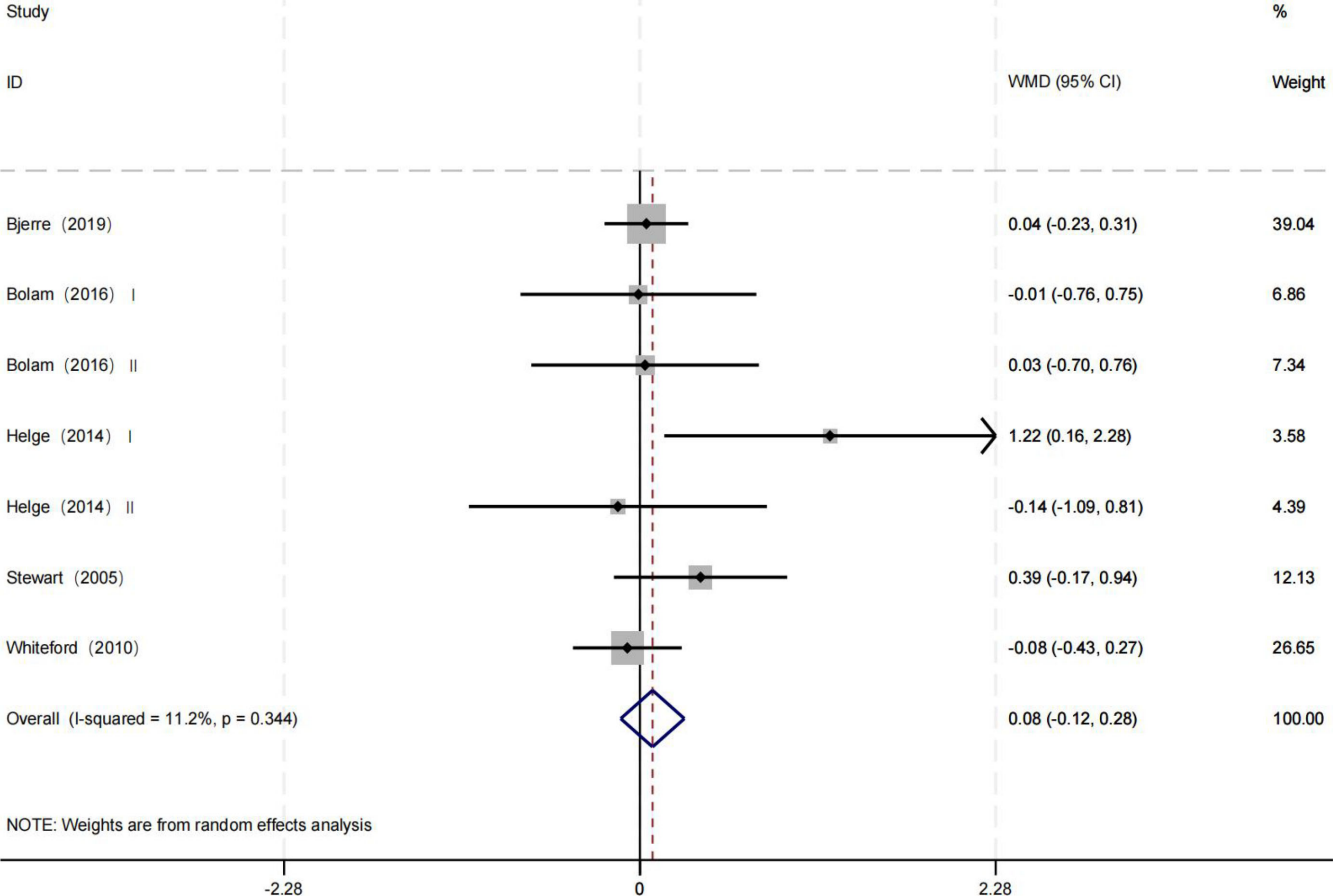
Meta-analysis results of the effect of exercise on whole body BMD.

Seven studies were included in the analysis. Exercise showed a mildly improved but no significant effect on total-body BMD (MD = 0.08, 95% CI −0.12 to 0.28), with zero heterogeneity (I² = 0%, P = 0.344). (**Figure 7**).

#### 3.4.6 Femoral shaft BMD

**Figure 8:**
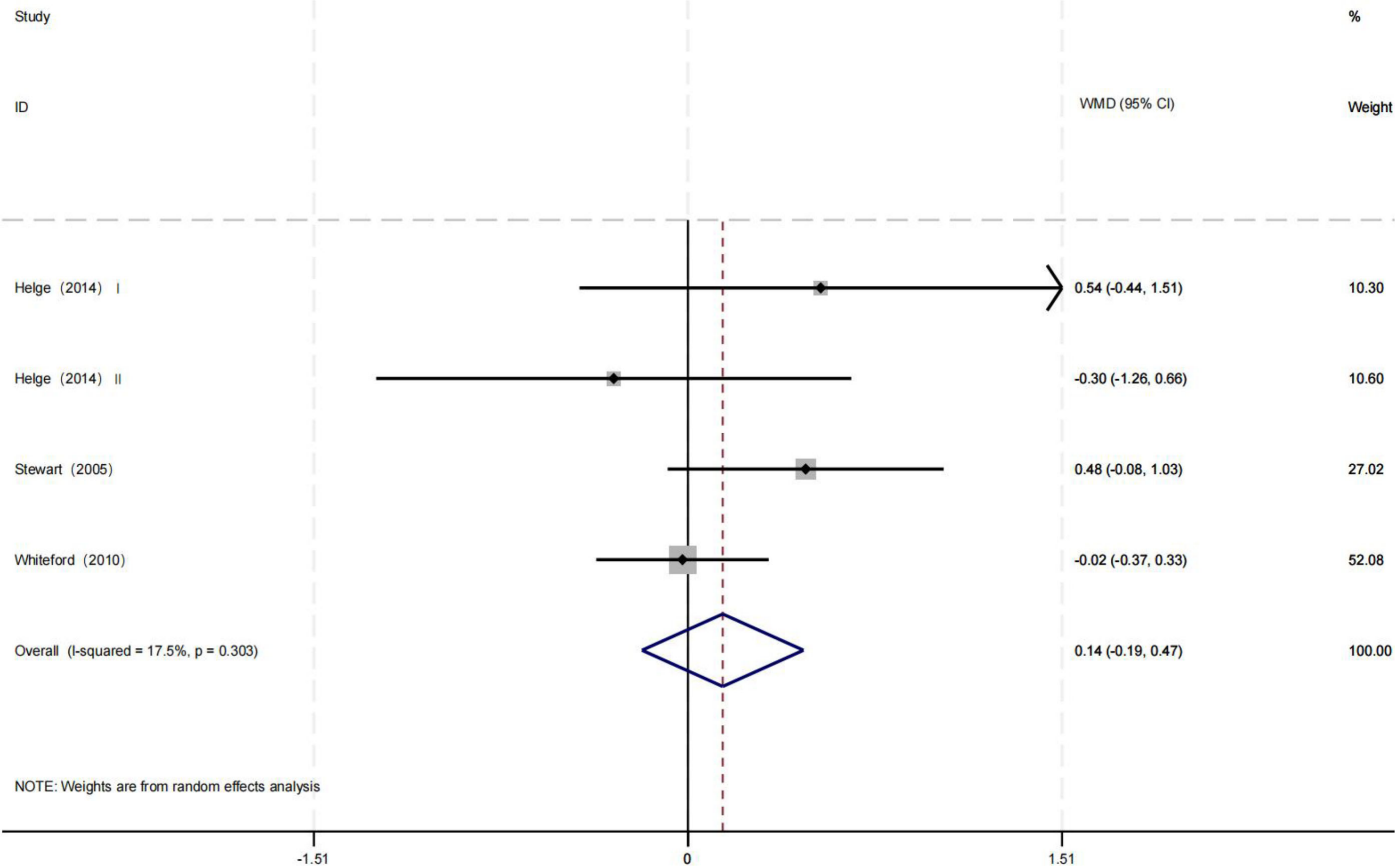
Meta-analysis results of the effect of exercise on femoral shaft BMD.

Four studies were included in this review. Exercise showed a mildly improved femoral shaft BMD, but the difference was not significant (MD = 0.02, 95% CI −0.14 to 0.28), with low heterogeneity (I² = 11.2%, p = 0.344). (**Figure 8**).

### 3.5 Sensitivity analyses

**Figure 9:**
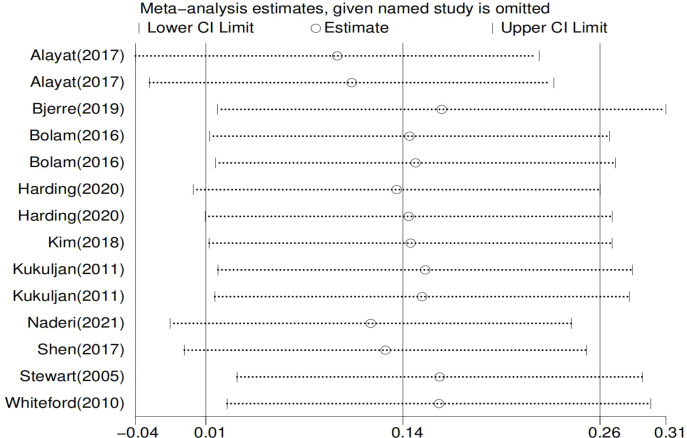
Sensitivity analysis plot for lumbar spine BMD.

**Figure 10:**
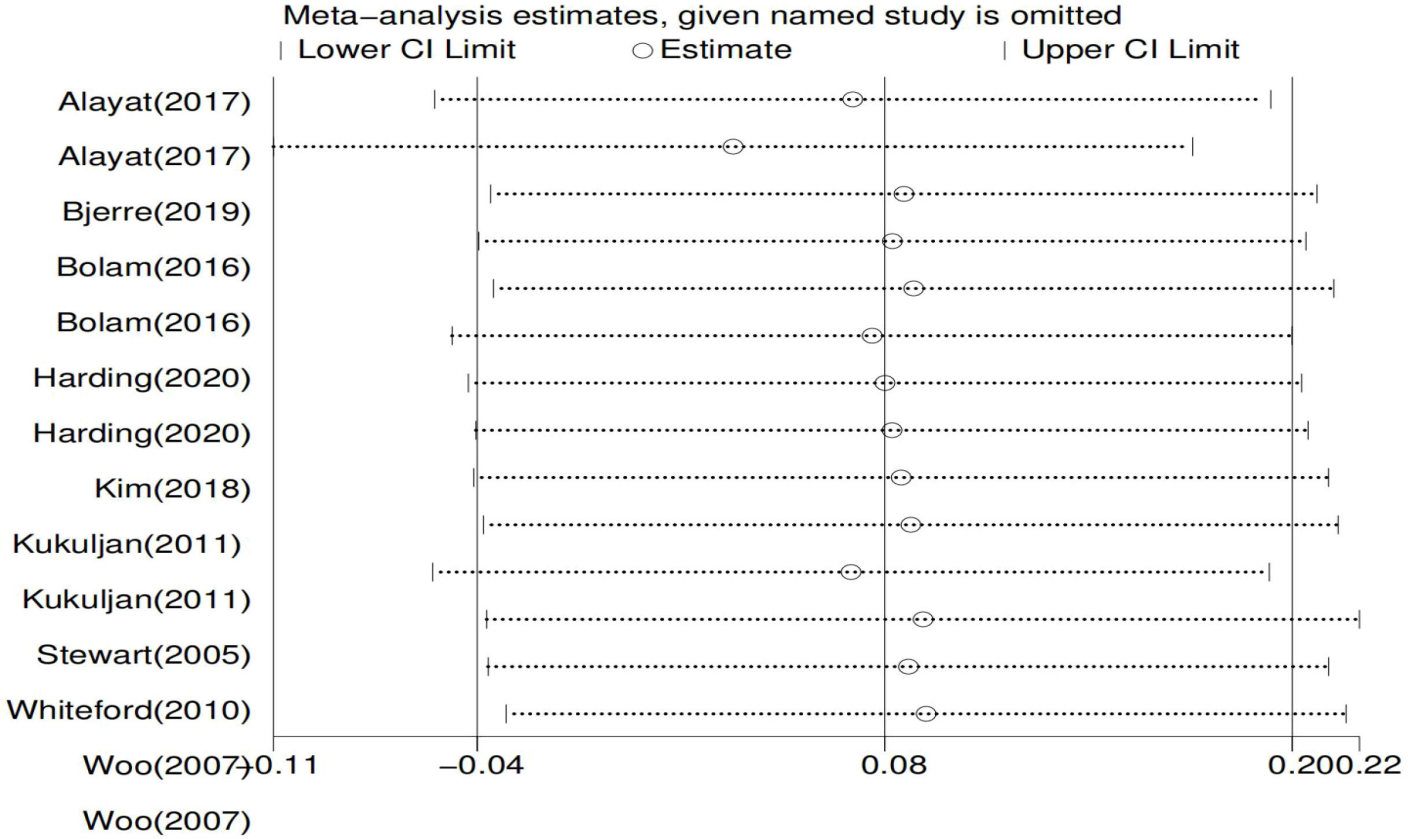
Sensitivity analysis plot for total hip BMD.

**Figure 11:**
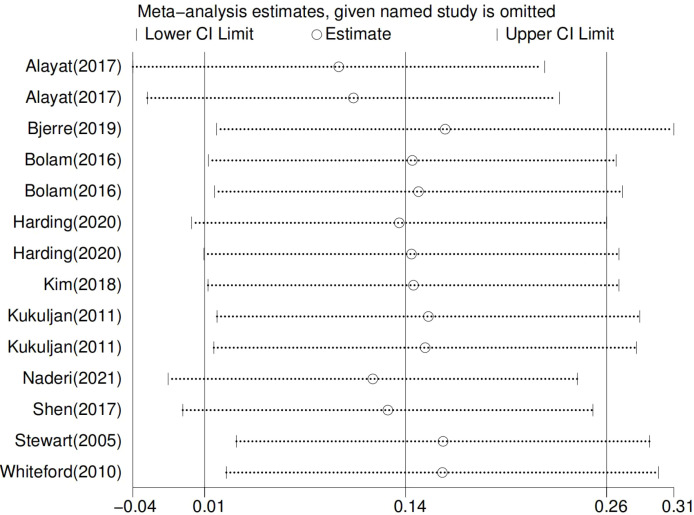
Sensitivity analysis plot for femoral neck BMD.

Sensitivity analyses were performed for all three primary outcomes to evaluate the stability of the synthesized results. The leave-one-out analysis demonstrated that the pooled effect sizes and their associated 95% confidence intervals remained consistent, with no single study exerting a disproportionate influence on the overall findings. These results confirm the robustness and reliability of the current meta-analysis (**Figures 9**–**11**).

### 3.6 Publication bias

**Figure 12:**
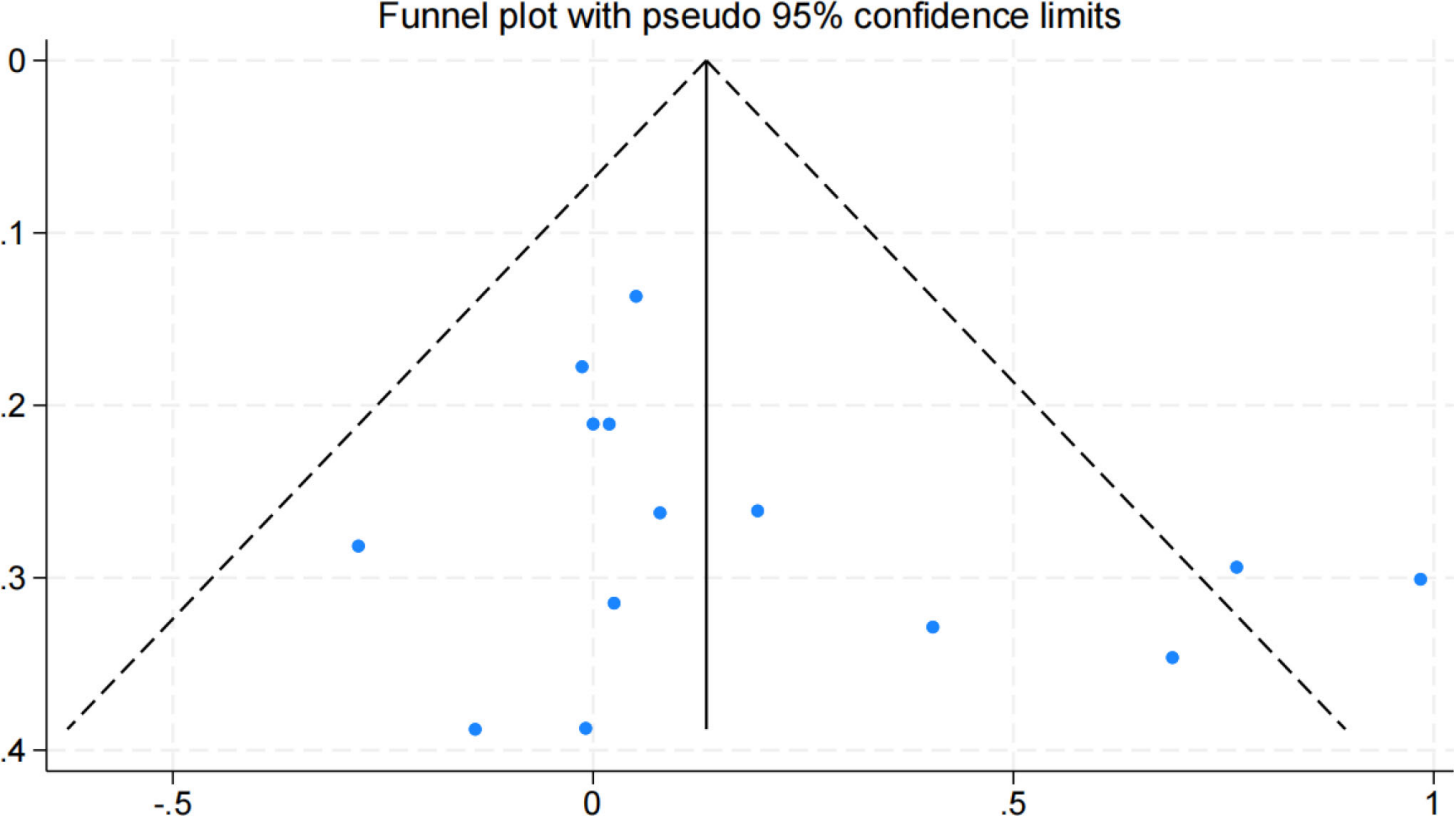
Funnel plot of the effect of exercise on lumbar spine BMD.

**Figure 13:**
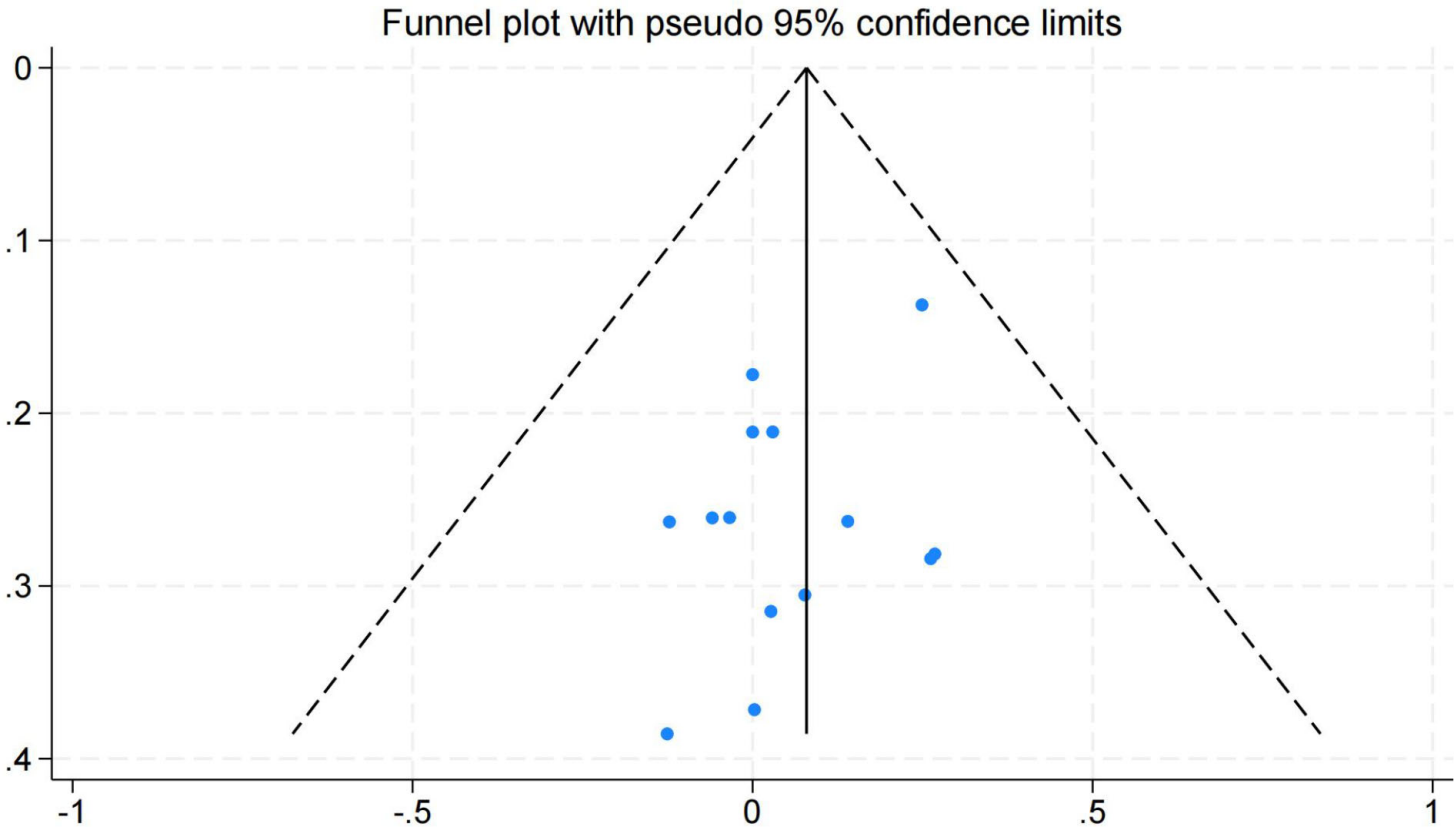
Funnel plot of the effect of exercise on total hip BMD.

**Figure 14:**
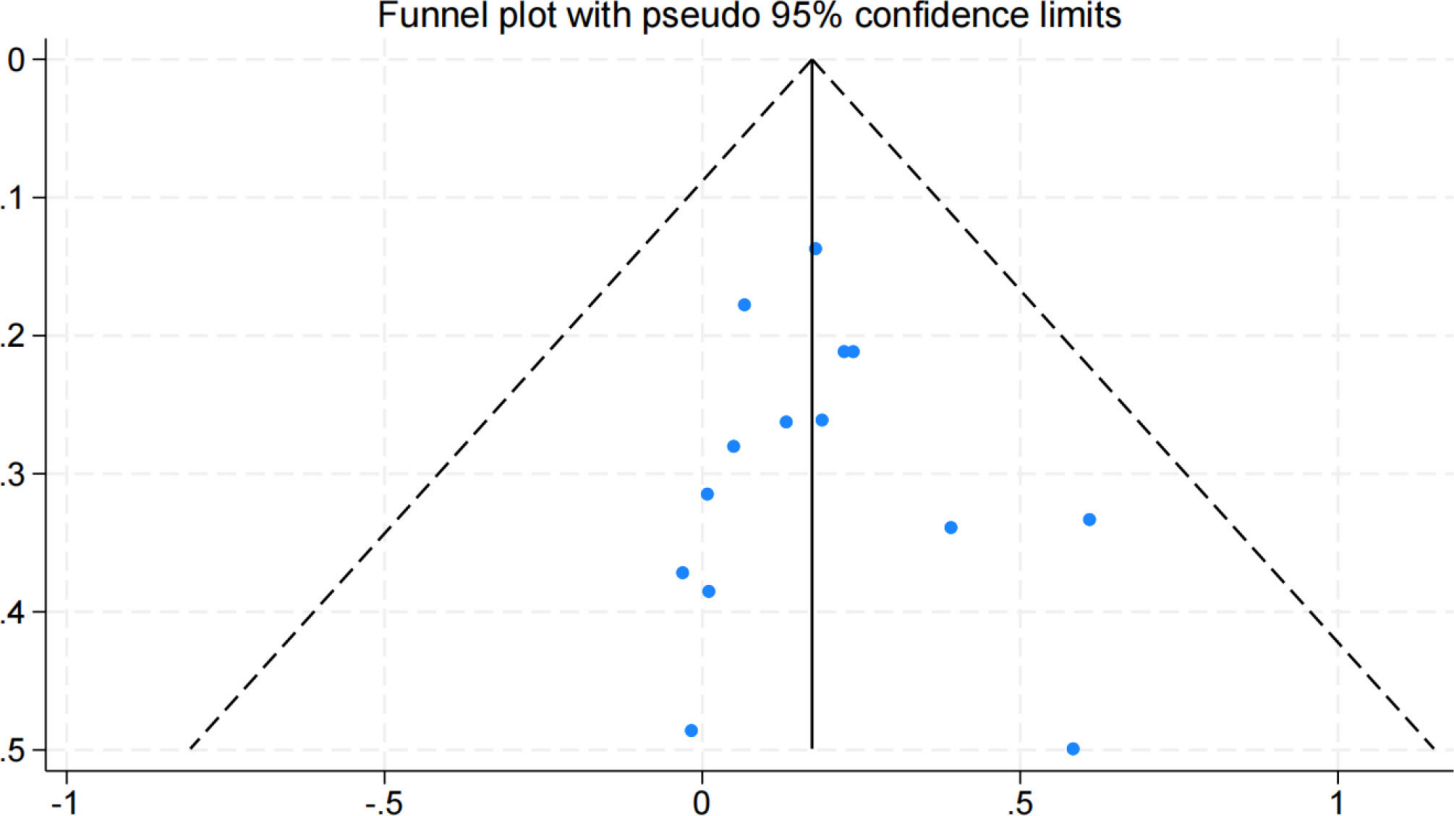
Funnel plot of the effect of exercise on femoral neck BMD. .

Funnel plots for the three primary outcomes appeared approximately symmetric, providing no visual evidence of publication bias (**Figures 12**–**14**). Publication bias was evaluated using Begg’s and Egger’s tests. The results indicated no evidence of significant publication bias for all assessed sites (lumbar spine: Begg p = 0.208, Egger p = 0.208; total hip: Begg p = 0.702, Egger p = 0.050; femoral neck: Begg p = 0.784, Egger p = 0.678). These findings suggest that the meta-analysis results are robust and not materially influenced by small-study effect.

### 3.7 Certainty of evidence

We evaluated the certainty of evidence for the primary outcomes using the GRADE framework. The GRADE assessment revealed moderate-certainty evidence for the increase in lumbar spine and femoral neck BMD, with both outcomes being downgraded by one level due to risk of bias. Conversely, the certainty of evidence for total hip BMD was rated as low, primarily downgraded for both risk of bias and imprecision. (**Table 4**).

**Table 4:** GRADE assessment.

Certainty assessment							№ of patients		Effect		Certainty	Importance
№ of studies	Study design	Risk of bias	Inconsistency	Indirectness	Imprecision	Other considerations	Exercise	Comparision	Relative (95% CI)	Absolute (95% CI)		
Lunar spine BMD												
14	randomised trials	serious	not serious	not serious	not serious	none	463	447	–	MD **0.13 g/cm2 higher** (0.01 higher to 0.26 higher)	⨁⨁⨁◯ Moderate	CRITICAL
Total hip BMD												
14	randomised trials	serious	not serious	not serious	serious	none	488	467	–	MD **0.08 g/cm2 higher** (0.05 lower to 0.2 higher)	⨁⨁◯◯ Low	CRITICAL
Femoral neck BMD												
14	randomised trials	serious	not serious	not serious	not serious	none	481	463	–	MD **0.17 g/cm2 higher** (0.04 higher to 0.03 higher)	⨁⨁⨁◯ Moderate	CRITICAL

CI, confidence interval; MD, mean difference. The bold values represent the results of the corresponding outcomes.

### 3.8 Subgroup analyses

**Figure 15:**
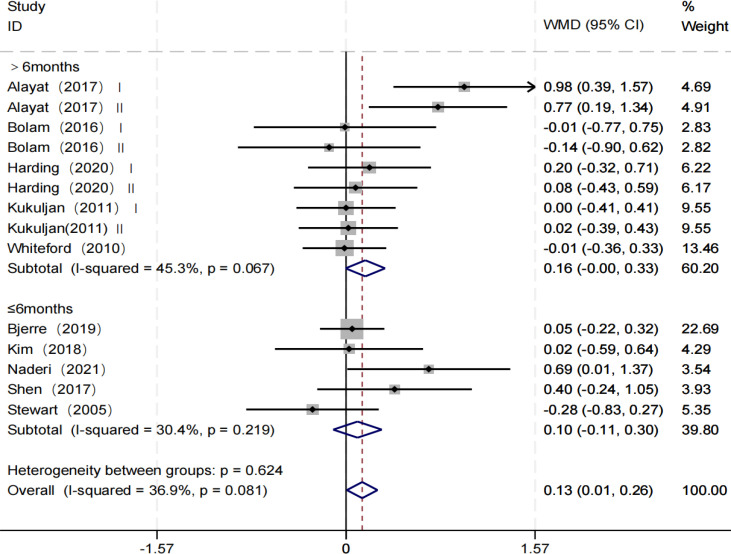
Subgroup analysis of lunar spine BMD by different intervention cycles.

**Figure 16:**
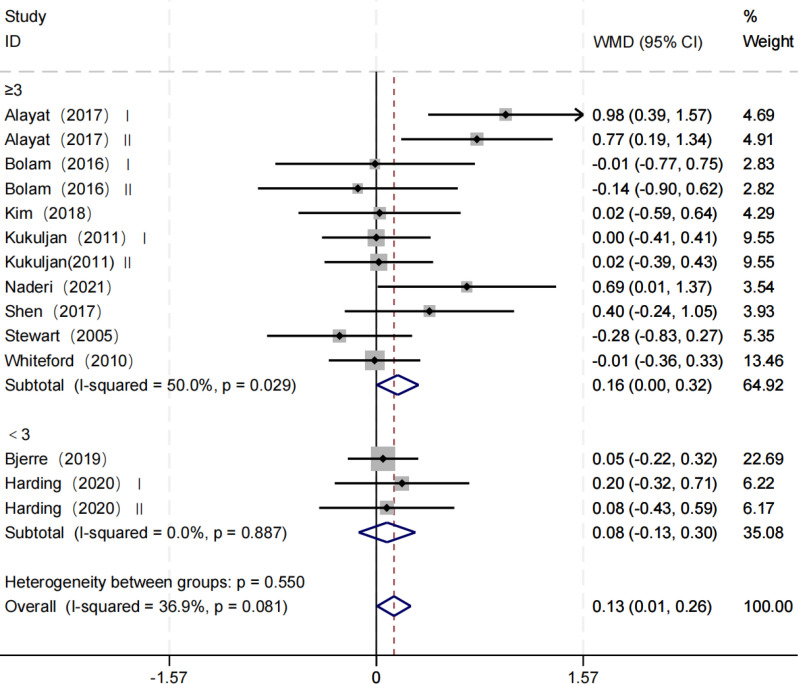
Subgroup analysis of lunar spine BMD by different intervention frequencies.

**Figure 17:**
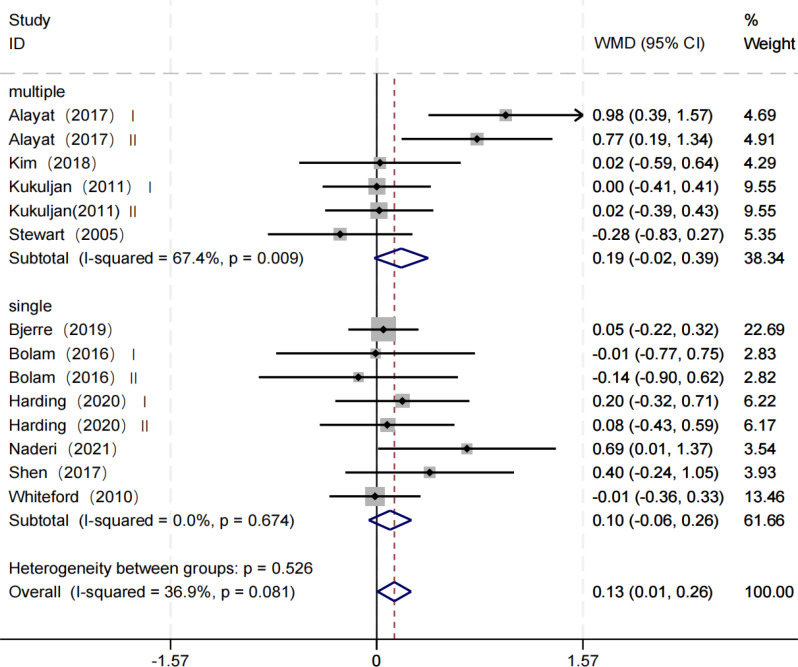
Subgroup analysis of lumbar BMD by modality of interventions (single VS multiple).

**Figure 18:**
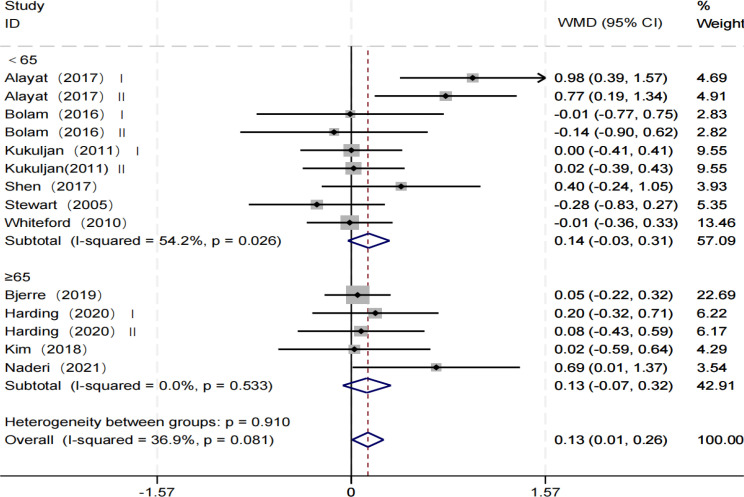
Subgroup analysis of lumbar BMD by patients’ age.

**Figure 19:**
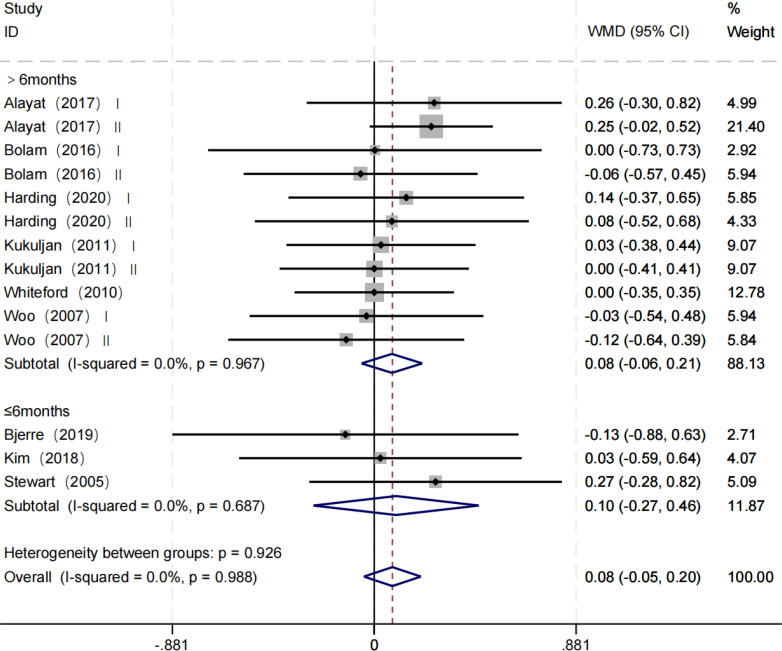
Subgroup analysis of total hip BMD by different intervention cycles.

**Figure 20:**
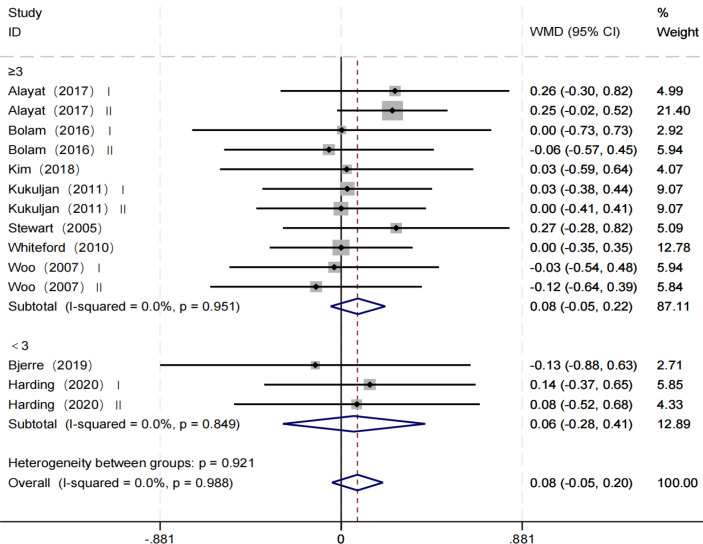
Subgroup analysis of total hip BMD by different intervention frequencies.

**Figure 21:**
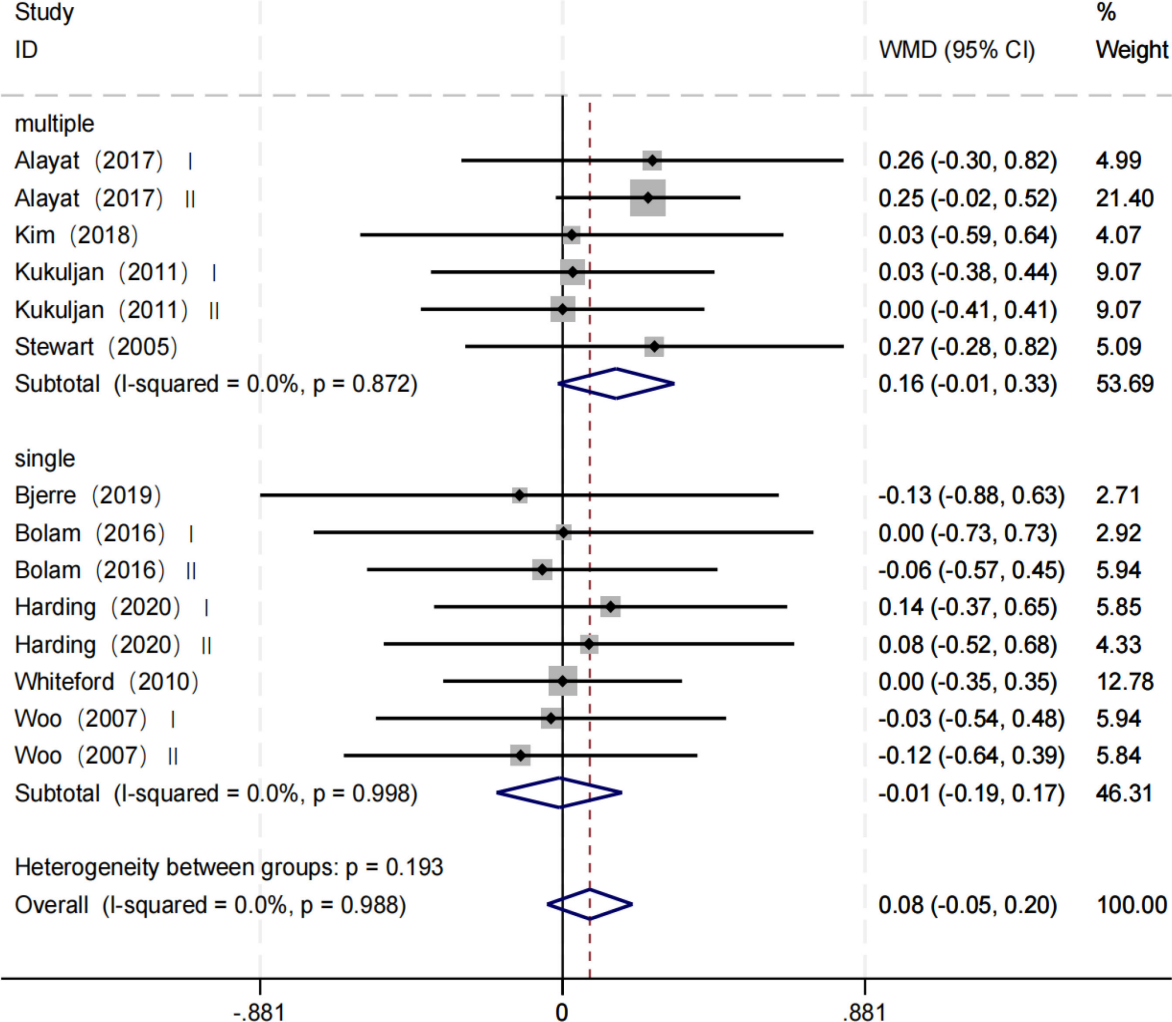
Subgroup analysis of total hip by modality of interventions (single VS multiple).

**Figure 22:**
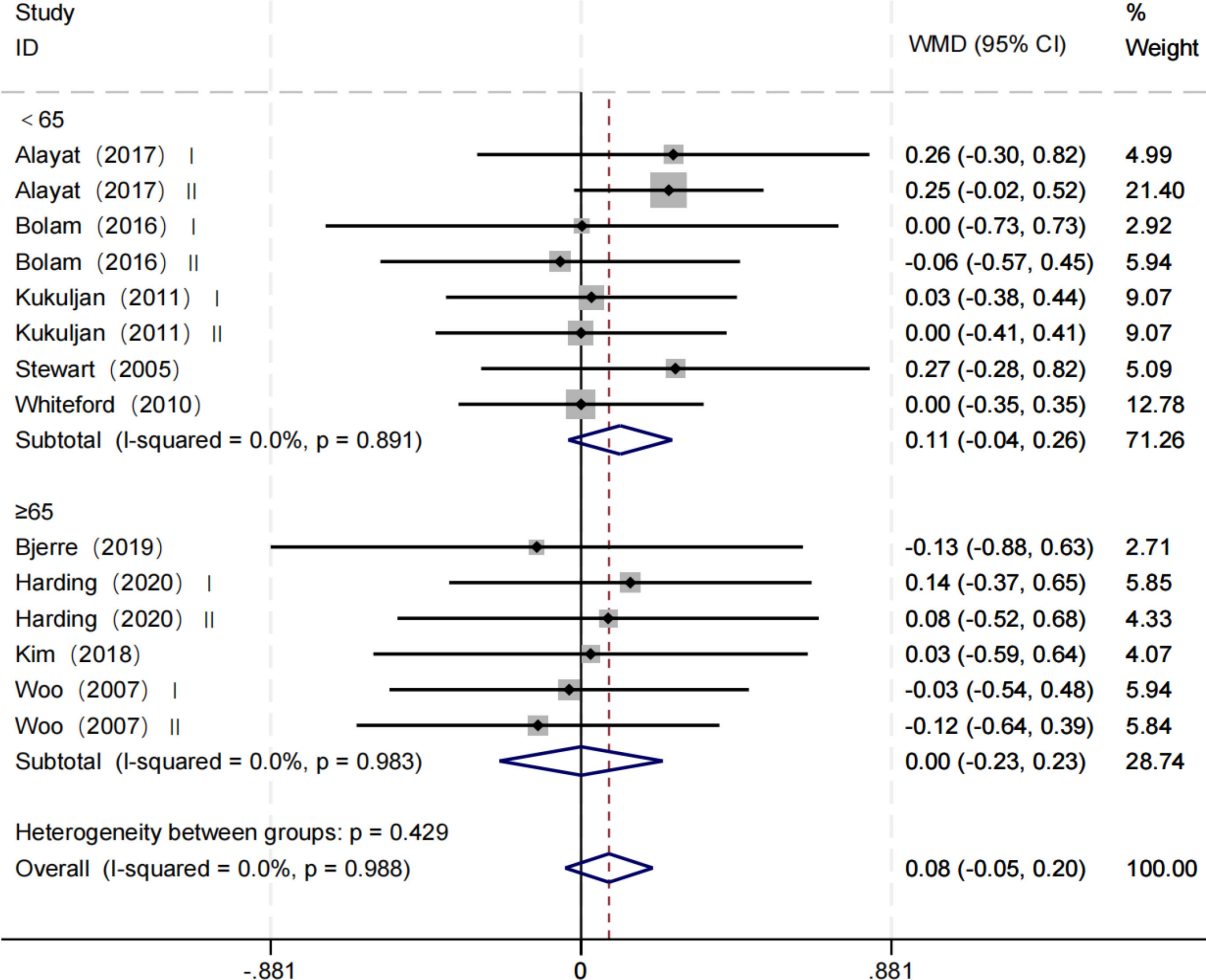
Subgroup analysis of total hip by modality of patients’ age.

**Figure 23:**
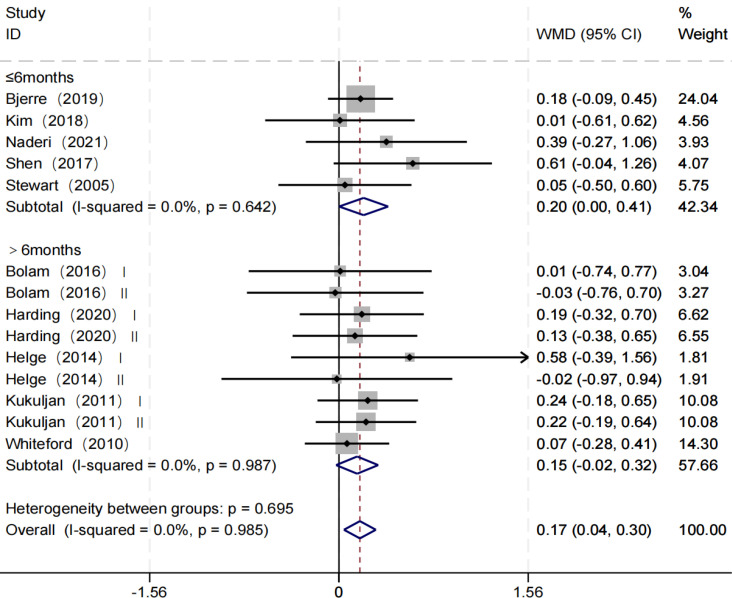
Subgroup analysis of femoral neck BMD by different intervention cycles.

**Figure 24:**
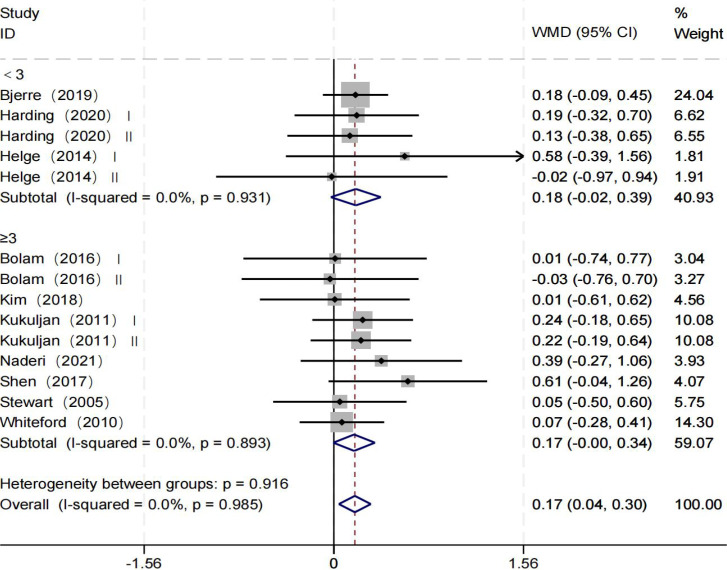
Subgroup analysis of femoral neck BMD by different intervention cycles.

**Figure 25:**
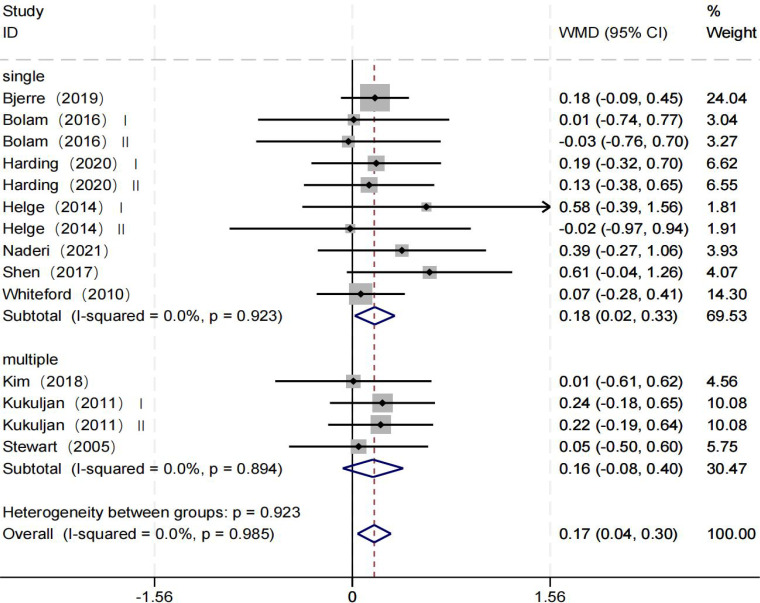
Subgroup analysis of femoral neck by modality of interventions (single VS multiple).

**Figure 26:**
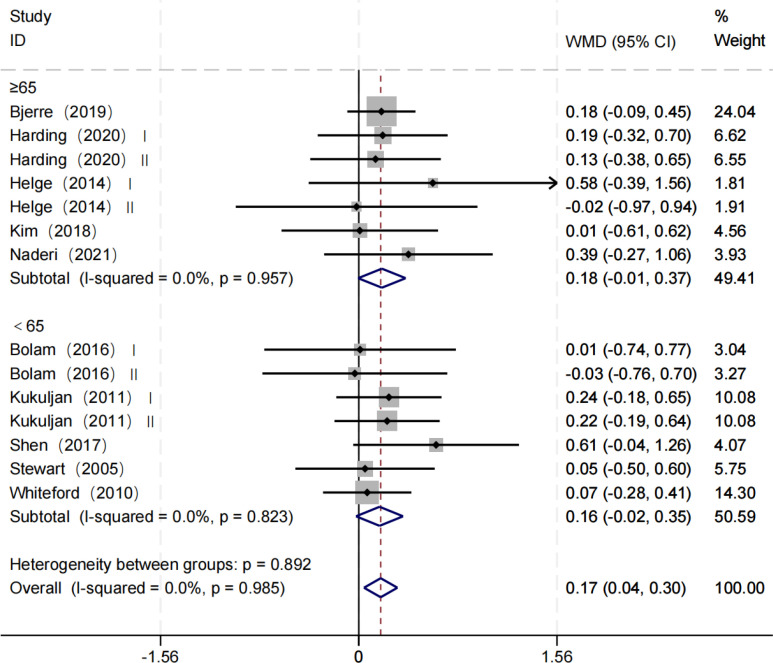
Subgroup analysis of femoral neck by modality of patients’ age.

Subgroup analyses revealed the robust and consistent beneficial effects of exercise interventions across diverse stratification factors. Age-stratified analyses (<65 vs. ≥s. years) showed no significant between-subgroup differences for lumbar spine, total hip, or femoral neck BMD. Notably, for total hip BMD, participants aged <65 years exhibited a numerically greater improvement than those aged ≥65 years. For lumbar spine BMD, a positive dose-response trend was identified: interventions of longer duration (>6 months: MD = 0.16, 95% CI: 0.00 to 0.33) and higher weekly frequency (re sessions/week: MD = 0.16, 95% CI: 0.00 to 0.32) produced numerically larger benefits than shorter or less frequent training. In addition, multicomponent exercise showed a higher mean improvement than single-modality exercise (0.19 vs. 0.10), suggesting that sustained and varied mechanical loading may better enhance lumbar spine bone mass. For total hip BMD, no significant between-subgroup differences were observed, although multicomponent exercise displayed a trend toward greater BMD gains. For femoral neck BMD, significant early responsiveness was found, with a substantial MD of 0.20 (95% CI: 0.00 to 0.41) in interventions lasting ≤6 months (**Figures 15**–**26**).

## 4 Discussion

This meta-analysis of 12 trials demonstrated that exercise intervention significantly improved BMD at the lumbar spine (MD = 0.16) and femoral neck (MD = 0.15) in middle-aged and older men, whereas the improvement in total hip BMD did not reach statistical significance (MD = 0.09).

The lumbar spine and femoral neck are high-risk sites for osteoporotic fractures (L. Li et al., 2024). The present findings indicate that exercise interventions have a beneficial effect on BMD at these two sites. From a biomechanical perspective, mechanical loading applied to bone tissue drives interstitial fluid flow through the canaliculi and lacunae, generating shear stress at the cellular level and deforming the osteocyte membrane. These changes initiate bone remodeling, promoting alternating cycles of bone resorption and formation (Rochefort et al., 2010). Recent studies have elucidated the molecular mechanisms underlying this process. Mechanical stimuli can induce osteocytes to produce signaling molecules such as nitric oxide (NO), prostaglandin E2 (PGE2), and adenosine triphosphate (ATP), thereby modulating the balance between osteoblast and osteoclast activity (Wang et al., 2022). Simultaneously, the Wnt/β-catenin pathway is activated via the Lrp5/6–Frizzled receptor complex, upregulating osteogenic gene expression and enhancing bone formation (Robling and Turner, 2009). In addition, mechanical stretching can transmit signals through the integrin–FAK/MAPK pathway, driving osteoblast proliferation and differentiation (X. Li et al., 2021). During exercise, muscle contractions not only provide external mechanical loading but also exert paracrine effects on bone via the secretion of myokines, such as IGF-1 (Hong and Kim, 2018).

Exercise did not exert a statistically significant effect on the total hip BMD in men. The total hip region contains a relatively large proportion of cortical bone, which is structurally dense, has a low turnover rate, and responds slowly to mechanical loading. In contrast, trabecular bone, with its higher surface area-to-volume ratio and richer blood supply, is more metabolically active and sensitive to mechanical stimuli (Bennell et al., 2010). When a measurement site encompasses both cortical and trabecular compartments, exercise-induced alterations in bone metabolism may be diluted, resulting in a smaller net change in overall bone mineral density at the site (Napoli et al., 2012; Haderslev et al., 2000), which may consequently lead to an effect that does not reach statistical significance. Similar patterns were observed at other skeletal sites, where the femoral shaft and greater trochanter showed only mild and non-significant improvements. Similarly, whole body BMD exhibited only slight changes. Overall, exercise-induced adaptations were more pronounced in trabecular-rich regions such as the lumbar spine and femoral neck, while cortical-dominated areas showed only minimal changes within the study periods of the included trials.

Compared with previous meta-analyses conducted in male populations, the present study provides a more systematic understanding of the skeletal site-specific responses to mechanical loading. The significant improvement observed in lumbar spine BMD is consistent with the findings of most related studies, with discrepancies only noted in a few reports (Hamilton et al., 2022; Ashe et al., 2021). Such divergence may be attributed to the larger sample size in the present study, which conferred greater ability to detect the relatively modest intervention effects in men. The significant benefit detected in femoral neck BMD further confirms the high responsiveness of this site to exercise intervention, aligning with the mainstream consensus in the literature. In contrast, no significant improvement was found in total hip BMD, which differs from the small but significant benefits reported in some studies (Lu et al., 2023; Mages et al., 2021; Ashe et al., 2021). This finding lends support to the biological hypothesis that the hip region, abundant in cortical bone, is inherently less responsive to mechanical loading in men. Furthermore, this study provides novel insights into exercise dose-response relationships. Contrary to previous conclusions that interventions lasting 12 months or longer are required to produce effects, our subgroup analysis identified significant early improvements in femoral neck BMD with short-term interventions of six months or less (Lu et al., 2023). We also confirmed the central role of training frequency of three sessions per week or more and multicomponent exercise regimens in optimizing bone mass accrual, filling the gaps in male-specific exercise prescription parameters not elaborated in prior research.

Comparison between the present study and meta-analyses in postmenopausal women reveals that exercise yields shared BMD improvements at the lumbar spine and femoral neck across both populations. However, whereas postmenopausal women exhibit a widespread skeletal response across multiple sites including the total hip, middle-aged and older men demonstrate more modest and localized gains, with limited effects on the overall hip region (Winters-Stone et al., 2012; Hejazi et al., 2025). A large body of epidemiological and clinical evidence has shown that, in women, circulating estrogen levels decline sharply after menopause and remain low (Santoro et al., 2001; Sowers et al., 2008; Randolph et al., n.d.) Estrogen plays a dominant role in the regulation of bone metabolism in women (Khosla and Monroe, 2018; Riggs et al., 2002) and the abrupt postmenopausal fall in estrogen induces a high bone turnover state (Cheng et al., 2022) in which exercise interventions more readily accelerate bone formation and produce relatively pronounced gains in BMD (Dalsky, 1990).

In males, the preservation of BMD is predominantly governed by testosterone and its aromatization to estradiol, which synergistically modulate osteoblastic and osteoclastic activity (Khosla and Monroe, 2018; Finkelstein et al., 2013). Unlike the abrupt postmenopausal decline observed in women, age-related decreases in testosterone and estradiol levels in men are more gradual (Dalsky, 1990; Wu et al., 2008; Harrall et al., 2025), and the associated changes in bone metabolism are relatively modest, reflecting a lower turnover state compared with that in women. Under these conditions, the bone-remodeling response elicited by exercise is less marked than that in women, particularly at predominantly cortical sites, such as the total hip, making it more difficult to detect statistically significant improvements over a relatively short intervention. The nonsignificant effect of exercise on total hip BMD in middle-aged and older men observed in the present study is likely to reflect the combined influence of these sex-specific endocrine differences and structural characteristics of the hip region.

Subgroup analyses further clarified the influence of exercise parameters on skeletal adaptation. Higher training frequency (≥3 sessions/week) and longer intervention duration (>6 months) were associated with greater improvements in lumbar spine BMD. These findings support the time-dependent and cumulative nature of bone adaptation, whereby sustained mechanical loading is required to effectively stimulate bone formation. Additionally, multicomponent exercise demonstrated superior efficacy over single-modality interventions, likely due to the application of diverse mechanical stimuli that enhance osteogenic signaling and reduce adaptive desensitization (Hamilton et al., 2022). Notably, femoral neck BMD exhibited clear early responsiveness within 6 months of intervention, indicating site-specific temporal adaptation patterns. Although no significant differences were observed between age groups (<65 vs. ≥65 years), younger individuals exhibited a tendency toward greater total hip BMD improvement. This may be related to age-associated declines in bone turnover and mechanosensitivity, indicating that older adults may require more intensive or targeted loading strategies to achieve comparable skeletal benefits.

## 5 Strengths and limitation

This systematic review and meta-analysis has several notable strengths. First, it focuses on middle-aged and older men, a population that is often underrepresented in osteoporosis research compared with postmenopausal women, thereby providing valuable evidence to inform exercise-based clinical strategies. Second, subgroup analyses based on key exercise parameters, including frequency, duration, and modality, provide important insights into the dose–response relationship between mechanical loading and site-specific BMD adaptations. Third, the minimal to low heterogeneity observed in hip-related outcomes, including total hip, femoral neck, and greater trochanter, strengthens the robustness and reliability of the pooled estimates for these skeletal regions. Nevertheless, several limitations should be considered. Despite including 12 trials, the overall sample size remains modest, which may limit the power to detect subtle changes in cortical-rich regions such as the femoral shaft. In addition, methodological concerns in some studies, particularly in randomization and allocation concealment, reduced the certainty of evidence for the primary outcomes. Finally, the lack of follow-up beyond 18 months limits conclusions on the long-term effects of exercise on fracture prevention, underscoring the need for high-quality randomized controlled trials with extended follow-up.

## 6 Conclusions

This systematic review and meta-analysis demonstrates that exercise interventions induce site-specific improvements in bone mineral density in middle-aged and older men. The most consistent benefits are seen at the lumbar spine and femoral neck, which are clinically high-risk sites for osteoporotic fractures. Although changes in bone mineral density at the total hip and other cortical-rich regions did not reach statistical significance, an overall positive adaptive trend in the skeleton was observed. Subgroup analyses indicate that a training frequency of ≥f sessions per week, an intervention duration of >6 months, and multicomponent exercise are key factors for optimizing the osteogenic effects of exercise. Notably, the femoral neck may show a relatively earlier response to mechanical loading.

Overall, exercise serves as a non-pharmacological intervention with favorable tolerability and clinical practicality for preserving skeletal health in this population. Future research with large sample sizes and long follow-up periods is needed to clarify the long-term impact of exercise interventions on reducing fracture risk.

## Data Availability

The original contributions presented in the study are included in the article/supplementary material. Further inquiries can be directed to the corresponding author.
